# Exercise training reduces ventricular arrhythmias through restoring calcium handling and sympathetic tone in myocardial infarction mice

**DOI:** 10.14814/phy2.13972

**Published:** 2019-02-25

**Authors:** Rujie Qin, Nobuyuki Murakoshi, DongZhu Xu, Kazuko Tajiri, Duo Feng, Endin N. Stujanna, Saori Yonebayashi, Yoshimi Nakagawa, Hitoshi Shimano, Akihiko Nogami, Akira Koike, Kazutaka Aonuma, Masaki Ieda

**Affiliations:** ^1^ Department of Cardiology Faculty of Medicine Graduate School of Comprehensive Human Sciences University of Tsukuba Tsukuba Japan; ^2^ Department of Internal Medicine (Endocrinology and Metabolism) Faculty of Medicine University of Tsukuba Tsukuba Japan; ^3^ Medical Science Faculty of Medicine University of Tsukuba Tsukuba Japan

**Keywords:** Autonomic nervous system, calcium handling, exercise, myocardial infarction, ventricular tachycardia

## Abstract

Exercise can improve morbidity and mortality in heart failure patients; however, the underlying mechanisms remain to be fully investigated. Thus, we investigated the effects of exercise on cardiac function and ventricular arrhythmias in myocardial infarction (MI) induced heart failure mice. Wild‐type male mice underwent sham‐operation or permanent left coronary artery ligation to induce MI. MI mice were divided into a sedentary (MI‐Sed) and two intervention groups: MI‐Ex (underwent 6‐week treadmill exercise training) and MI‐βb (oral bisoprolol treatment (1 mg/kg/d) without exercise). Cardiac function and structure were assessed by echocardiography and histology. Exercise capacity and cardiopulmonary function was accepted as oxygen consumption at peak exercise (peak VO_2_). Autonomic nervous system function and the incidence of spontaneous ventricular arrhythmia were evaluated via telemetry recording. mRNA and protein expressions in the left ventricle (LV) were investigated by real‐time PCR and Western blotting. There were no differences in survival rate, MI size, cardiac function and structure, while exercise training improved peak VO_2_. Compared with MI‐Sed, MI‐Ex, and MI‐βb showed decreased sympathetic tone and lower incidence of spontaneous ventricular arrhythmia. By Western blot, the hyperphosphorylation of CaMKII and RyR2 were restored by exercise and β‐blocker treatment. Furthermore, elevated expression of miR‐1 and decreased expression of its target protein PP2A were recovered by exercise and β‐blocker treatment. Continuous intensive exercise training can suppress ventricular arrhythmias in subacute to chronic phase of MI through restoring autonomic imbalance and impaired calcium handling, similarly to that for β‐blockers.

## Introduction

Despite important advances in drug and device therapy, myocardial infarction (MI) remains a leading cause of death and morbidity worldwide, especially because of heart failure and ventricular tachyarrhythmia. Acute ischemic injury leads to the activation of neurohormones and cytokines, subsequent myocardial remodeling, a further decline in cardiac function, and finally overt heart failure (Schober and Knollmann 2007; Zhang et al. 2010). During this pathophysiological progress, malignant arrhythmias such as ventricular tachyarrhythmias, associated with imbalance of the autonomic nervous system, are a major cause of death (Shen and Zipes 2014; Kalla et al. 2016).

There are increasing evidences that exercise training is an effective non‐pharmacological treatment for cardiovascular diseases with a beneficial effect on disease progression and survival (La Rovere et al. 2002; Anderson et al. 2016). The previous studies reported that exercise could beneficially reduce post‐MI remodeling and improve intracellular calcium handling (Lu et al. 2002; Kemi et al. 2007; French et al. 2008; Guizoni et al. 2016). Exercise training also causes a reverse remodeling of cardiac autonomic regulation such that parasympathetic activity is enhanced while sympathetic activity is reduced, resulting to improvement of heart rate variability (HRV) (Malfatto et al. 1996) and suppression of ventricular tachyarrhythmia (Billman 2009; Kukielka et al. 2011; Bonilla et al. 2012). Recently, intensive exercise training has been reported to have greater improvement than low intensive or moderate intensive training in cardiac rehabilitation course (Jaureguizar et al. 2016; Howden et al. 2018). Although most studies showed beneficial effects of exercise training in MI patients and animals, it remains to be fully elucidated what are the cellular/molecular mechanisms that link alterations in cardiac autonomic regulation and increased risk for arrhythmias and how does exercise training alter these cellular/molecular abnormalities to protect against malignant arrhythmias after MI.

Thus, in this study we explored the effects and mechanisms by intensive exercise training in post‐MI mice, especially anti‐arrhythmic potential, via comparing the effects of exercise versus an established first‐line medicine β‐blocker treatment (Yancy et al. 2013).

## Materials and Methods

### Ethics statement

All animal experimental procedures in this study were approved by the Institutional Animal Experiment Committee of the University of Tsukuba. Experiments were conducted in accordance with the Guide for the Care and Use of Laboratory Animals published by the US National Institutes of Health and the Fundamental Guideline for Proper Conduct of Animal Experiment and Related Activities in Academic Research Institutions under the jurisdiction of the Ministry of Education, Culture, Sports, Science and Technology of Japan.

### Experimental protocol

A total of 96 wild‐type male mice (C57Bl/6J) purchased from CLEA Japan, Inc. (Tokyo, Japan) were used in this study. After adapting to the environment for 2−3 weeks, 75 11−12‐week‐old mice received MI operation by permanent ligation of the proximal left anterior descending coronary artery, as described previously (Stujanna et al. 2017). In the sham group, 21 mice were subjected to a similar procedure without arterial ligation. In the first week after the MI operation, 20 mice died. A final 55 MI mice were then randomly divided into the sedentary group (20 mice; MI‐Sed), exercise training group (17 mice; MI‐Ex), and β‐blocker treatment group (18 mice; MI‐βb).

### Exercise training protocol

Mice in the MI‐Ex group received 6‐week treadmill training starting on the 8th day after the MI operation. Training was performed with a 10‐lane treadmill (MK‐680; Muromachi Kikai Co., Ltd., Tokyo, Japan). The training intensity was determined as the maximum speed and endurance time when the animals were unable to run further despite receiving electrical stimuli. According to the previous researches (Speaker et al. 2014; Rolim et al. 2015), 6‐week intervention was decided to obtain enough effectiveness. In the first week, the training intensity started at 6 m per min (m/min) for 30 min, and increased progressively in the following days until reaching 18 m/min for 45 min/day when mice showed exhaustion. This maximal intensity (18 m/min for 45 min, at 0% slope) was maintained for 5 weeks, 5 days per week.

### β‐blocker treatment protocol

MI‐βb mice received 6 weeks of Bisoprolol hemifumarate (1 mg/kg/day; Tokyo Chemical Industry Co., Ltd., Tokyo, Japan) treatment via water solution from the 8th day after the MI operation (Yamada et al. 2014). To deliver the correct dosage, we first evaluated water intake per day of each mouse after the operation (approximately 4 mL/day), and then the appropriate concentration was determined (0.0075 mg/mL).

### Echocardiographic assessment of left ventricular function

We performed echocardiographic assessments three times during the experiment (on 7th day after MI/sham operation, after 3 weeks of intervention and after 6 weeks of intervention), as described previously (Stujanna et al. 2017). In brief, mice were anesthetized by isoflurane, and echocardiographic images were obtained at the papillary muscle level using a 40‐MHz transducer connected to a Doppler echocardiographic system (Vevo 2100; Visual Sonics, Toronto, Canada). Both the parasternal long‐axis view and short‐axis view were acquired. Left ventricular (LV) end‐diastolic diameter (LVDd), LV end‐systolic diameter (LVDs), LV ejection fraction (LVEF) (%), and fractional shortening (FS) (%) were measured or calculated using short‐axis two‐dimensional M‐mode images.

### Assessment of exercise capacity and cardiopulmonary function

A metabolic running wheel chamber connected to a mass spectrometer for respiratory analysis (ARCO 2000; ARCO system Inc., Chiba, Japan) was used to assess exercise capacity and cardiopulmonary function. After 6 weeks of the different interventions, 5–6 animals in each group were randomly chosen and weighed before examination. Mice were placed individually inside the sealed running wheel, forced to run at gradually increasing speeds, and maintained at the last velocity until exhaustion. Oxygen consumption at peak exercise (peak VO_2_, NmL/min/kg) was determined and normalized by body weight in kilograms. Exercise capacity and cardiopulmonary function were accepted as peak VO_2_ (NmL/min/kg) (Iwase et al. 2004).

### Electrocardiography telemetry and heart rate variability analysis

Continuous 24‐h ECG recordings were obtained from implanted telemetry devices. After 6 weeks of intervention, randomly‐selected mice (*n* = 5–6/group) underwent telemetry implantation. Mice were anesthetized with a mixture of ketamine and xylazine, and the tele‐transmitters (ETA‐F20; Data Sciences International DSI, St. Paul, MN) were subcutaneously implanted into the midline of the back. After implantation, each mouse was housed in a single cage, and allowed to recover for several days before starting the measurement. An analog signal from the telemetric receiver was applied to the ECG and autonomic nervous system (ANS) modulations. Data were collected and analyzed off‐line by a commercially‐available software (Dataquest A.R.T. Data Sciences International DSI). Premature ventricular contractions (PVC) and ventricular tachycardias (VT: more than three PVC beats in succession) were pick out and counted from ECG recordings. Heart rate variability (HRV) analysis is generally used to index ANS function. Mean R‐R intervals (in ms) and standard deviation of R‐R intervals (SDNN) in time‐domain analysis are considered to reflect total autonomic variability (Shaffer and Ginsberg 2017). In frequency‐domain analysis, the HF (high frequency power) band reflects parasympathetic activity, while the LF (low frequency power) band reflects the combined function of both sympathetic and parasympathetic activity. A high LF/HF (the ratio of low frequency power to high frequency power) indicates sympathetic dominance (Thireau et al. 2008).

### Tissue analysis

After the 6 week‐intervention, the hearts, lungs, and sartorius muscles were excised and weighed at postmortem. The LVs at the level of the papillary muscle were fixed with 4% paraformaldehyde, embedded in paraffin, sectioned into 4‐μm thick slices, and stained with Masson's trichrome. Cross‐section images of the LV were obtained by a digital microscope (Biozero BZ‐X700; Keyence, Osaka, Japan), and MI area was calculated by dividing the collagen depositing area to the total area. In non‐infarcted area, the fibrosis area was calculated by image analyzing software (ImageJ analysis software ver.1.45; NIH; Bethesda, MD). The anatomical ratios of tissue weights to body weight (BW) were calculated, including the ratios of heart weight to BW, LV weight to BW, right ventricular weight to BW, lung weight to BW, left atrial + right atrial weight to BW, and the sartorius muscle (on the femur) weight to BW.

### Real‐time PCR

We performed real‐time PCR to assess gene expression levels in the LVs, as described previously (Xu et al. 2012; Stujanna et al. 2017). In brief, total RNA was extracted from LV tissues using the RNeasy Fibrous Tissue Mini Kit (Qiagen, Hilden, Germany). A 1 μg of RNA was then reverse transcribed to cDNA with a High‐Capacity cDNA RT Kit (Thermo Fisher Scientific, Inc., Waltham, MA). The mRNA expression levels of the target genes were analyzed by an ABI Prism 7500 sequence detection system (Thermo Fisher Scientific) with gene‐specific primers and probe sets (Integrated DNA Technologies, Coralville, IA). The PCR mixture (10 μL total volume) consisted of the template, primer, and probe for each gene (250 nmol/L), and the PrimeTime Gene Expression Master Mix (Integrated DNA Technologies, Skokie, IL). PCR amplification was performed as follows: 1 cycle at 95°C for 10 min, and 40 cycles at 94°C for 15 sec and 60°C for 1 min. The primers and probes used in the study were as follows: *MHC‐α*(M00440359‐a1), *MHC‐β* (M01319005‐g1) (Thermo Fisher Scientific); *Nppb* (BNP: brain natriuretic peptide) (Mm.PT.58.8584045.g), *β1‐AR* (β1 adrenergic receptor; Mm.PT.58.41132658.g), *Grk5* (Mm.PT. 58.11422186), *β2‐AR* (β2 adrenergic receptor; Mm.PT.58.29310038.g), *Chrm2* (Mm.PT.58.42964182.g), *Col1a1* (Mm.PT.58.756.2513), *Col3a1* (Mm.PT.58.13848686), *TGF‐β1* (Rn01475964), *Atp2a2* (SERCA2a: sarcoplasmic reticulum calcium ATPase); Mm.PT.58.5303089), *PLN* (phospholamban; Mm.PT.58.43778023), and *RyR2* (ryanodine receptor type 2; Mm.PT.58.45974879) (Integrated DNA Technologies). For analyzing microRNAs (miRNA), 500 μg of RNA was reverse transcribed to cDNA with a miRNA primer using the miRNeasy Mini Kit (Qiagen). The miRNA primer assays used were miR‐1 (RT: 002064, PN4427975) and miR‐133a (TM: 000458, PN4427975). The PCR mixture was made and amplified as described above. The quantitative values of target mRNA and miRNA were normalized to expression of 18S rRNA (4319413E; Thermo Fisher Scientific). Data were obtained from three independent measurements (*n* = 8/group) performed in duplicate.

### Western blotting analysis

We evaluated protein expression in the LVs by Western blotting, as described previously (Xu et al. 2012; Stujanna et al. 2017). In brief, isolated LVs were homogenized in PRO‐PREP protein extraction solution (iNtRON Biotechnology, Inc., Kyungki‐Do, Korea), and the supernatants were collected. A 10 μg of proteins were transferred by semidry electroblotting from gels (Bio‐Rad Laboratory, Hercules, CA) to polyvinylidene difluoride membranes. The blots were then blocked with the primary antibodies as follows: phospho‐RyR2 (Ser 2814) (A010‐31; Badrilla, Leeds, UK), phospho‐RyR2 (Ser 2808) (A010‐30, Badrilla), RyR2 (PA5‐36121; Thermo Fisher Scientific), SERCA2a (2A7‐A1; Thermo Fisher Scientific), phospho‐Phospholamban (p‐PLN) (S16 + T17; ab62170; Abcam, Cambridge, UK), total Phospholamban (PLN) (ab2865; Abcam), phospho‐PKA (T197; ab75991; Abcam), PKA (ab187515; Abcam), phospho‐CaMKII (T286) (ab32678; Abcam), Oxidized‐CaMKII (Met281/282) (EMD Millipore, Darmstadt, Germany), total CaMKII (ab103840; Abcam), protein phosphatase 2A (PP2A)‐B56‐α (F‐10; sc‐271151; Santa Cruz, Dallas, TX), phospho‐Troponin‐I (p‐TNI) (Ser23/24) (#4004; Cell Signaling Technology, Danvers, MA), total Troponin‐I (TNI) (#4002; Cell Signaling Technology), PP1 (E‐9; sc‐7482; Santa Cruz), and β‐actin (4967s; Cell Signaling Technology). The blots were incubated with an appropriate horseradish peroxidase‐conjugated goat anti‐rabbit IgG (ab6721; Abcam) or horseradish peroxidase‐conjugated rabbit anti‐mouse IgG (ab97046; Abcam) secondary antibody. Immunoreactions were visualized with an enhanced chemiluminescence method (ECL Prime Western Blotting Detection; GE Healthcare, Southeast, UK). Densitometric analysis was performed on scanned immunoblot images (ImageJ analysis software ver.1.45; NIH; Bethesda, MD). Data were obtained from three independent measurements (*n* = 6/group).

### Statistics

All data are expressed as mean ± standard error of the mean (SEM). To compare values between the groups, continuous values were analyzed by one‐way ANOVA followed by post hoc‐testing with Bonferroni's test. The result for each experiment was obtained from three independent measurements. Significance was accepted when *P* < 0.05. Statistical analysis was performed using statistical software (IBM SPSS ver. 21.0; IBM Co. Ltd., Armonk, NY).

## Results

### Exercise training does not affect survival rate, MI size, and fibrosis in non‐infarcted area, but enhances exercise capacity and cardiopulmonary function

During the 6 weeks of training or treatment, three mice in MI‐Sed (3/20: 15.0%) and one mouse in MI‐Ex (1/17: 6.8%) died, while no mice died in Sham (0/21: 0.0%) and MI‐βb (0/18: 0.0%) groups. There was no significant difference in the survival rate between the MI groups. As ventricular rupture was not observed in the four mice that died, we assumed death from cardiac arrhythmia or heart failure.

**Figure 1:**
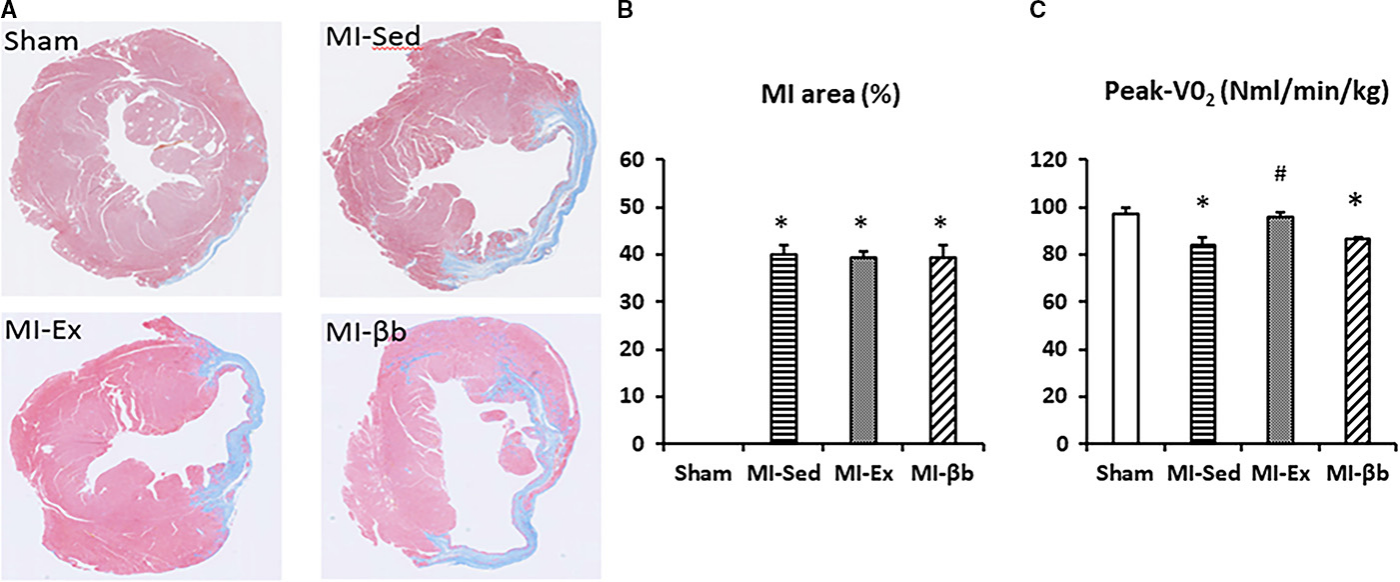
Cross‐sectional findings of the left ventricles at the papillary muscle level and cardiopulmonary function. (A) In Masson's trichrome staining images of the left ventricle (LV), the blue thinning region indicates the infarct area. (B) There was no difference in the infarct area between the three MI groups (MI‐Sed group: 40.1 ± 1.8% vs. MI‐Ex group: 39.4 ± 1.4% vs. MI‐βb: 39.3 ± 2.7%; *n* = 8/group). *P < 0.05, versus Sham; #*P* < 0.05, versus MI‐Sed. (C) Oxygen consumption at peak exercise (Peak‐VO_2_) was decreased in MI‐Sed and MI‐βb groups, and was restored in MI‐Ex, compared with Sham (Sham, 97.2 ± 2.3; MI‐Sed, 83.8 ± 3.7; MI‐Ex, 95.7 ± 2.4; MI‐βb, 86.3 ± 1.0, NmL/min/kg) (*n* = 5/group in Sham, MI‐Sed, and MI‐βb; *n* = 6 in MI‐Ex).

Masson's trichrome (MT) staining images of the LVs showed no differences in the infarct area between the three MI groups (Fig. 1A and B). We assessed the exercise capacity and cardiopulmonary function of mice via a metabolic running wheel chamber. Peak VO_2_ was decreased in MI‐Sed and MI‐βb compared with Sham, but was restored in MI‐Ex (Fig. 1C).

**Figure 2:**
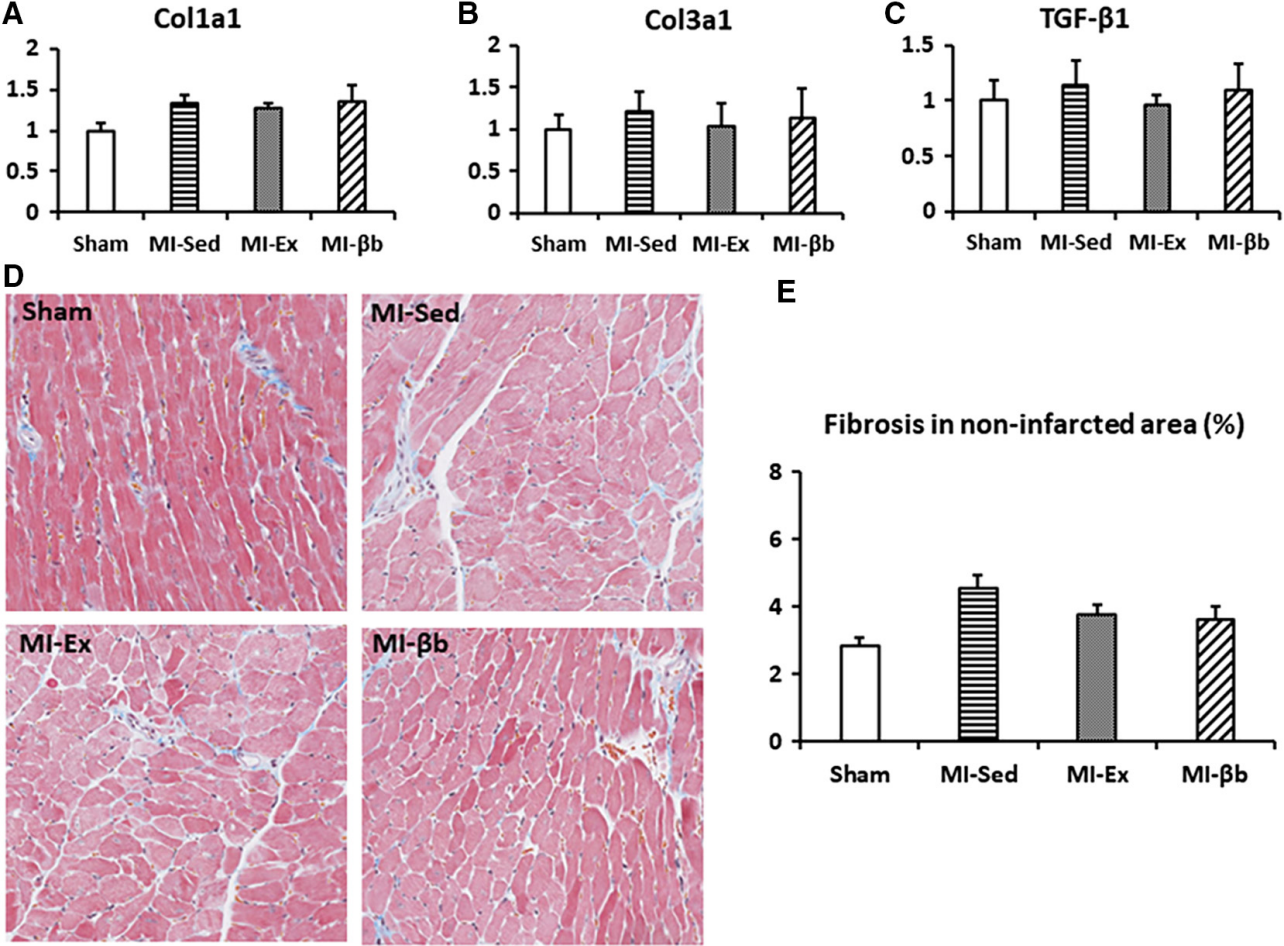
The fibrosis in non‐infarcted area of the left ventricles. (A, B and C) There were no significant differences in the gene expression levels of *Col1a1*,* Col3a1,* and *TGF‐β1* between all groups. (D) In Masson's trichrome staining images of non‐infarcted area (at 20 ×  magnification), the regions stained blue indicate collagen deposition. (E) There was no obvious difference in the collagen deposition area between groups in non‐infarcted area (Sham, 2.86 ± 0.21%; MI‐Sed, 4.53 ± 0.40%; MI‐Ex, 3.75 ± 0.67%; MI‐βb, 3.62 ± 0.40%; *n* = 6/group).

In the non‐infarcted area, real‐time PCR for the fibrosis markers showed no significant differences in the gene expression levels of *Col1a1*,* Col3a1,* and *TGF‐β1* (Fig. 2A, B, and C). MT staining images of the LVs showed there was no significant difference in the fibrosis area (Fig. 2C, D).

### Exercise training has no apparent effect on cardiac contractility or hypertrophy

**Figure 3:**
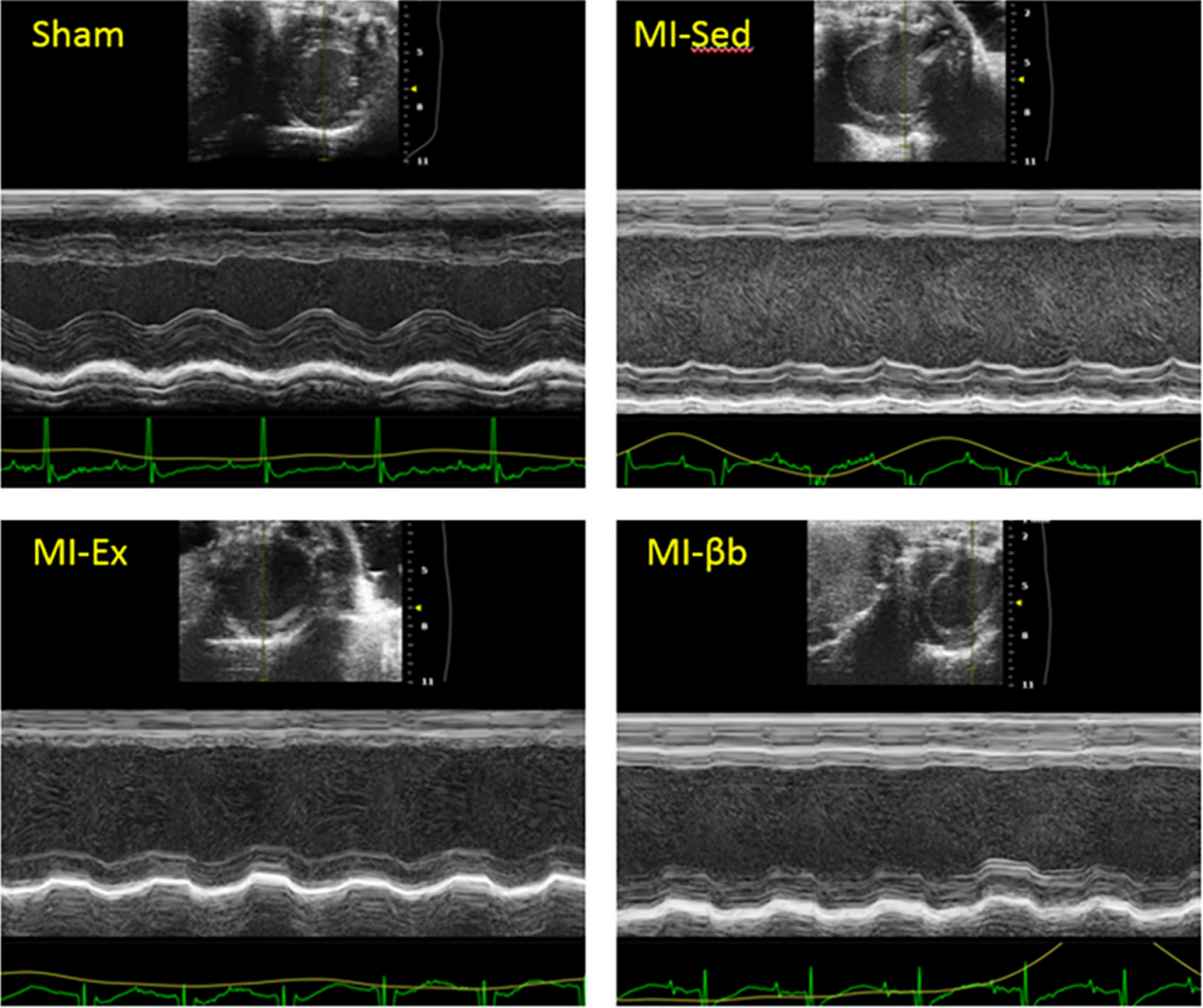
Representative images of echocardiographic analysis. The representative two‐dimensional M‐mode images of the left ventricular short‐axis view show thinning of the anteroseptal wall, enlargement of the left ventricle, and reduced left ventricular systolic function in all MI groups compared with Sham.

Before intervention, echocardiography at the 7th day after MI/sham operation demonstrated that LV was enlarged, and the LV systolic function was reduced in all MI groups compared with Sham, but there was no obvious difference between three MI groups. After 3‐week intervention, LVDd and LVDs in all MI mice were progressively increased; however, there were no significant differences between MI groups (Fig. 3). LVEF (%) and FS (%) were also not significantly different between MI groups. After 6‐week intervention, the results showed the similar tendency as 3 weeks after MI. LVEF (%) was slightly improved by exercise and β‐blocker treatment, while there were no significant differences in MI groups (Table 1).

**Table 1:** Echocardiographic analysis

Variable		Sham	MI‐Sed	MI‐Ex	MI‐βb
LVDd (mm)	pre	3.36 ± 0.13	4.54 ± 0.26*	4.62 ± 0.16*	4.81 ± 0.12*
	mid		4.83 ± 0.18	4.77 ± 0.16	4.94 ± 0.20
	post	3.49 ± 0.08	5.01 ± 0.10*	4.82 ± 0.17*	5.00 ± 0.13*
LVD s(mm)	pre	2.41 ± 0.16	3.95 ± 0.29*	4.02 ± 0.16*	4.17 ± 0.14*
	mid		4.16 ± 0.20	4.14 ± 0.17	4.27 ± 0.18
	post	2.45 ± 0.15	4.18 ± 0.17*	4.13 ± 0.18*	4.25 ± 0.12*
LVEF (%)	pre	56.12 ± 3.25	29.23 ± 2.91*	28.06 ± 1.32*	28.62 ± 1.85*
	mid		29.78 ± 2.40	28.45 ± 2.16	29.20 ± 1.68
	post	57.62 ± 3.10	31.03 ± 2.85*	30.91 ± 2.09*	31.49 ± 0.99*
FS (%)	pre	28.67 ± 2.06	13.67 ± 1.42*	13.05 ± 0.64*	13.41 ± 1.07*
	mid		14.03 ± 1.21	13.33 ± 1.11	13.73 ± 0.85
	post	28.65 ± 1.64	14.79 ± 1.40*	14.61 ± 1.08*	15.16 ± 0.57*

Data are presented as mean ± standard error of the mean (SEM). pre, at the 7th day after MI/sham operation; mid, after 3 weeks of intervention; post, after 6 weeks of intervention.

* *P* < 0.05, versus Sham.

**Table 2:** Anatomical data

Variable	Sham	MI‐Sed	MI‐Ex	MI‐βb
BW (g)	27.99 ± 0.54	29.36 ± 0.32	28.14 ± 0.46	29.18 ± 0.44
HW/BW (%)	4.01 ± 0.06	4.78 ± 0.12*	5.21 ± 0.15*	4.95 ± 0.13[Link]
LVW/BW (%)	2.98 ± 0.05	3.62 ± 0.08*	3.85 ± 0.11*	3.71 ± 0.10*
RVW/BW (%)	0.78 ± 0.03	0.86 ± 0.03	1.01 ± 0.05	0.94 ± 0.03
LW/BW (%)	4.58 ± 0.09	4.63 ± 0.09	5.01 ± 0.11	4.64 ± 0.06
AW/BW (%)	0.24 ± 0.01	0.28 ± 0.01	0.34 ± 0.02	0.31 ± 0.02
SMW/BW (%)	8.16 ± 0.19	7.30 ± 0.26	9.10 ± 0.28#	7.74 ± 0.32

Data are presented as mean ± SEM. BW, body weight; HW/BW (%), the ratio of whole heart weight to BW; LVW/BW (%), the ratio of left ventricular weight to BW; RVW/BW (%), the ratio of right ventricular weight to BW; LW/BW (%), the ratio of lung weight to BW; AW/BW (%), the ratio of left atrial + right atrial weight to BW; SMW/BW (%), the ratio of sartorius muscle (on the femur) weight to BW.

^*^ *P* < 0.05, versus Sham; ^#^ *P* < 0.05, versus MI‐Sed (*n* = 9/group).

Anatomical data indicated that the ratios of LV weight to BW, right ventricular weight to BW, and lung weight to BW were significantly increased in all MI groups compared with Sham (Table 2). These values also showed a trend towards a slight increase after 6 weeks. However, only the ratio of the sartorius muscle weight to BW was significantly increased in MI‐Ex compared with the others. These data suggest that exercise training strengthened the skeletal muscles but had no apparent effect on cardiac hypertrophy.

### Exercise regulates the autonomic imbalance and reduces the occurrence of ventricular arrhythmias

**Figure 4:**
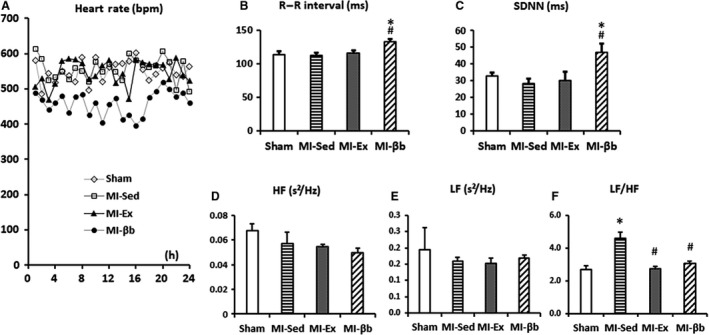
Heart rate variability (HRV) analysis in the time and frequency domains. (A) Mean heart rate was significantly lower in MI‐βb compared with the other groups (Sham, 550 ± 17 beats per min (bpm); MI‐Sed, 563 ± 51 bpm; MI‐Ex, 554 ± 29 bpm; MI‐βb, 476 ± 35 bpm). (B, C) In time‐domain parameters of HRV analysis, MI‐βb demonstrated prolonged mean R–R intervals (Sham, 114.1 ± 4.2 ms; MI‐Sed, 112.2 ± 3.8 ms; MI‐Ex, 115.4 ± 4.1 ms; MI‐βb, 132.9 ± 3.6 ms) and standard deviation of R–R intervals (SDNN; Sham, 32.6 ± 2.0 ms; MI‐Sed, 28.0 ± 3.0 ms; MI‐Ex, 29.8 ± 5.4 ms; MI‐βb, 46.7 ± 5.2 ms). (D) The high frequency power (HF) in frequency domain analysis showed a trend towards a decrease in MI groups (Sham, 0.068 ± 0.0059 s²/Hz; MI‐Sed, 0.057 ± 0.0096 s²/Hz; MI‐Ex, 0.057 ± 0.0021 s²/Hz; MI‐βb, 0.050 ± 0.0035 s²/Hz); (E) The low frequency power (LF) has no obvious difference (Sham, 0.19 ± 0.07 s²/Hz; MI‐Sed, 0.16 ± 0.01 s²/Hz; MI‐Ex, 0.15 ± 0.002 s²/Hz; MI‐βb, 0.17 ± 0.01 s²/Hz). (F) The LF/HF ratio, indicating the sympathetic tone activity, was significantly increased in MI‐Sed compared with Sham, and it was restored in MI‐Ex and MI‐βb (Sham, 2.60 ± 0.90 s²/Hz; MI‐Sed, 3.92 ± 0.38 s²/Hz; MI‐Ex, 2.83 ± 0.14 s²/Hz; MI‐βb, 3.68 ± 0.27 s²/Hz), (*n* = 4/group). *P < 0.05, versus Sham; #*P* < 0.05, versus MI‐Sed.

Heart rate was significantly lower in the MI‐βb group compared with the other groups (Fig. 4A). In time‐domain parameters, MI‐βb mice demonstrated prolonged mean R–R intervals and SDNN (Fig. 4B and C). Frequency domain analysis indicated that HF and LF showed no significant differences while LF/HF, an index of sympathetic tone activity, was decreased in MI‐Ex and MI‐βb compared with MI‐Sed (Fig. 4D, E, and F).

**Figure 5:**
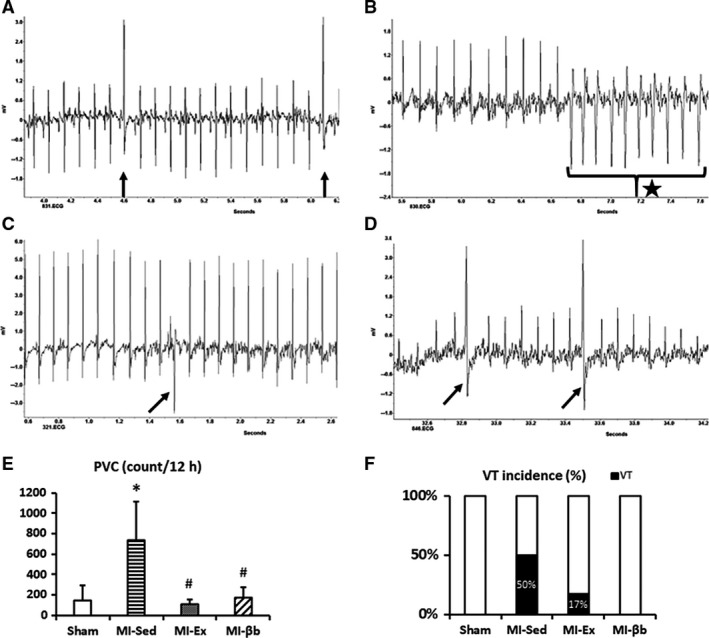
Representative images of the spontaneous ventricular arrhythmic events including premature ventricular contractions (PVC) and ventricular tachycardias (VT) in every group (The arrow indicates PVC, and the star indicates VT). (A) in Sham; (B) in MI‐Sed; (C) in MI‐Ex; (D) in MI‐βb. (E) Mice in MI‐Sed showed an increased incidence of PVCs compared with Sham, while exercise training and β‐blocker treatment effectively reduced the occurrence (Sham, 148 ± 143 counts/12 h; MI‐Sed, 739 ± 377 counts/12 h; MI‐Ex, 109 ± 44 counts/12 h; MI‐βb, 172 ± 102 counts/12 h, *n* = 6/group). (F) VTs were frequently observed in 3 of 6 mice (50%) in MI‐Sed and 1 of 6 mice (17%) in MI‐Ex. The longest duration of VT was 22 seconds in a MI mouse. However, no VT was observed in Sham and MI‐βb (*n* = 6/group). *P < 0.05, versus Sham; #*P* < 0.05, versus MI‐Sed.

Next, we calculated the occurrence of spontaneous ventricular arrhythmic events including PVCs and VTs (Fig. 5A–D) in continuous ECG recordings. Mice in MI‐Sed showed an increased incidence of PVCs compared with Sham, while exercise training and β‐blocker treatment effectively reduced the occurrence (Fig. 5E). Notably, VTs were frequently observed in 3 of 6 mice (50%) in MI‐Sed and the longest duration of VT was 22 sec in a MI mouse, while 1 of 6 mice (17%) in MI‐Ex, and no VT was observed in Sham or MI‐βb (Fig. 5F). These data suggest that chronic exercise training can reduce the episodes of spontaneous ventricular arrhythmias, similarly to β‐blocker treatment.

### Exercise influences calcium handling‐related molecules and regulates activity of kinases and phosphatases

**Figure 6:**
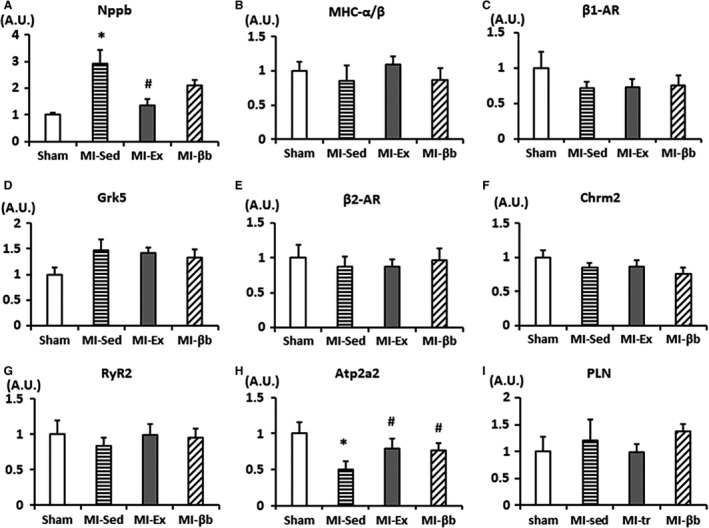
Gene expression levels analyzed by real‐time PCR. (A) The mRNA expression of *Nppb* (BNP) was significantly higher in MI‐Sed than Sham, and was significantly lower in MI‐Ex and MI‐βb than MI‐Sed. (B–F) The expression levels of *MHC α/β, β1‐AR*,* Grk5, β2‐AR,* and *Chrm2* showed no obvious changes. (G–I) In calcium handling related genes, there were no obvious differences in the expressions of *Ryr2* and *PLN*, while *Atp2a2* (*SERCA2a*) was significantly lower in MI‐Sed than Sham, and was restored in MI‐Ex and MI‐βb (*n* = 8/group). *P < 0.05, versus Sham; #*P* < 0.05, versus MI‐Sed.

Next, we assessed the expression of relevant genes in LV tissues. The expression of *Nppb*, which encodes BNP, was significantly higher in MI‐Sed versus Sham, but significantly lower in MI‐Ex versus MI‐Sed (Fig. 6A). The ratio of *MHC α/β* showed no obvious change (Fig. 6B). The expression level of β1‐AR was slightly decreased, and the expression of Grk5 was slightly increased in MI‐Sed compared to Sham. However, we couldn't find the significances between groups (Fig. 6C and D). The expressions of Chrm2 and β2‐AR also showed no apparent changes (Fig. 6E and F).

The calcium handling pathway is highly involved in the incidence of cardiac arrhythmias and determines the force of myocardium contraction. RyR2 releases calcium from (SR) to the cytoplasm, while SERCA2a and PLN, an inhibitor of SERCA2a, coordinately mediate calcium reuptake into the SR (Lanner et al. 2010). Therefore, we investigated the changes in expressions of calcium handling related genes. The mRNA expressions of *RyR2* and *PLN* were comparable in all groups, while the mRNA expression of *Atp2a2* (SERCA2a) was significantly decreased in MI‐Sed compared with Sham, and it was significantly increased in MI‐Ex and MI‐βb groups (Fig. 6G, H and I).

**Figure 7:**
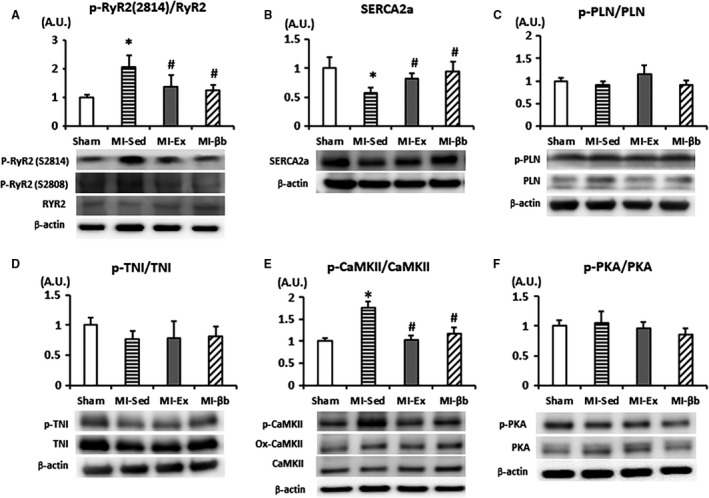
Western blotting analysis. (A) The phosphorylated RyR2 at Serine 2814 site was significantly increased in MI‐Sed compared with Sham and was decreased in MI‐Ex and MI‐βb. There was no change in the expression of phosphorylated RyR2 at Serine 2808 site or total RyR2. (B) The protein expression of SERCA2a in MI‐Sed was significantly decreased compared with Sham and was recovered in MI‐Ex and MI‐βb. (C) The expression of p‐PLN/PLN showed slightly increased in MI‐ Ex, but the differences were not significant between all groups. (D) Although both the expressions of TNI and p‐TNI were decreased in MI groups compared with Sham, the ratio of p‐TNI to TNI showed no obvious difference. (E) The expression level of p‐CaMKII was obviously increased in MI‐Sed, and it was decreased in MI‐Ex and MI‐βb without significant alterations of ox‐CaMKII and total CaMKII expressions. (F) There were no significant differences in PKA and phosphorylated PKA (T197) expressions between all groups (*n* = 6/group). *P < 0.05, versus Sham; #*P* < 0.05, versus MI‐Sed.

Next, we assessed the protein expressions of calcium handling related molecules. The ratio of phosphorylated RyR2 at Serine 2814 to total RyR2 was significantly increased in MI‐Sed compared with Sham and decreased in MI‐Ex and MI‐βb groups (Fig. 7A). By contrast, there were no differences in the ratio of phosphorylated RyR2 at Serine 2808 to total RyR2 between the MI groups. The expression of SERCA2a was significantly decreased in MI‐Sed compared with Sham, and restored in MI‐Ex and MI‐βb groups (Fig. 7B). The expression of p‐PLN/PLN showed slightly increased in MI‐ Ex, but the difference was not significant between all groups (Fig. 7C). It has been reported that reduced p‐TNI could be an indicator of increased Ca^2+^ sensitivity in post‐MI (van der Velden et al. 2004). In our study, although both the expressions of TNI and p‐TNI were decreased in MI groups compared with Sham, the ratio of p‐TNI to TNI showed no obvious difference (Fig. 7D).

We further determined the protein activity of the upstream kinases that regulate the amplitude and kinetics of calcium cycling. Exercise and β‐blocker treatment suppressed CaMKII hyperphosphorylation without alteration of expression levels of oxidized‐CaMKII and total CaMKII (Fig. 7E). By contrast, there were no significant differences in PKA and phosphorylated PKA (T197) expressions (Fig. 7F).

### Exercise decreases miR‐1 expression and improves PP2A expression

**Figure 8:**
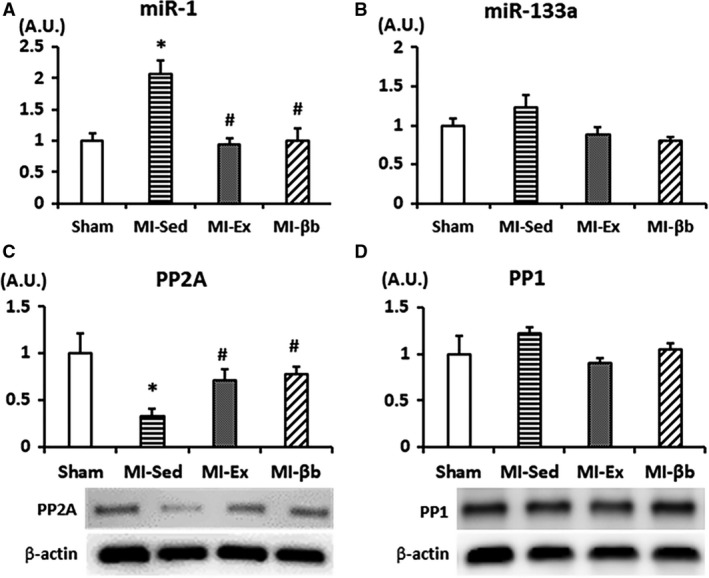
(A) The expression of microRNA‐1 (miR‐1) in the LV was elevated in MI‐Sed, and reduced by exercise and β‐blocker treatment. (B) There was no difference in the expression of miR‐133a between the groups (*n* = 5/group). (C) There was no difference in expression of protein phosphatase 1 (PP1), while PP2A‐B56‐α (F‐10) expression was significantly decreased in MI‐Sed compared to that in Sham and reversed in MI‐Ex and MI‐βb (D) (*n* = 6/group) *P < 0.05, versus Sham; #*P* < 0.05, versus MI‐Sed.

The miRNAs are a class of small single‐stranded and highly conserved non‐coding RNAs, which post‐transcriptionally regulate gene expression (Bartel 2009). In the miRNA family, miR‐1and miR‐133a were the first described striated muscle‐specific miRNAs and are closely linked to cardiac disorders such as MI, heart failure, and arrhythmia (Matkovich et al. 2009; Belevych et al. 2011). Therefore, we evaluated the expression of miR‐1 and miR‐133a. We found that miR‐1 expression was significantly elevated in MI‐Sed, but restored by exercise and β‐blocker treatment (Fig. 8A), while there was no significant difference in miR‐133a expression (Fig. 8B).

Protein phosphorylation is regulated by the balance of kinase and phosphatase activities. PP1 and PP2A are major serine–threonine phosphatases involved in the dephosphorylation of specific substrates (Terentyev et al. 2009). The expression of the PP2A regulatory subunit B56α (F‐10) was significantly decreased in MI‐Sed, and restored in MI‐Ex and MI‐βb groups (Fig. 8C). However, there were no differences in PP1 protein expression between the groups (Fig. 8D).

## Discussion

In this study, we found that chronic intensive exercise training improved the imbalance of sympathetic and parasympathetic activities, reduced the incidence of PVC and VT, and restored calcium handling in MI mice, despite no obvious alterations in cardiac structure or function. Thus, exercise training in subacute to chronic phase of MI did not increase the risk of malignant arrhythmias, but rather restores autonomic function and cardiac electrical stability.

Although both exercise and treatment with β‐blockers did not alter MI area and cardiac systolic function, we found that exercise training significantly improved peak VO_2_. This finding is consistent with the previous observation that exercise has a better effect on cardiopulmonary function and exercise capacity (Jaureguizar et al. 2016). We also found that 6‐week exercise restored autonomic imbalance showed by LF/HF and decreased spontaneous ventricular arrhythmias. Autonomic nervous remodeling occurs after MI, which is critical for the incidence of tachyarrhythmias and sudden cardiac death (Shen and Zipes 2014). Billman GE and colleagues reported that β2‐adrenergic stimulation was relatively activated, and its receptor antagonist could suppress the incidence of VF in canine MI model through restoring intracellular Ca^2+^ transients (Billman et al. 1997). They also reported that endurance exercise training normalized repolarization and calcium‐handling abnormalities, prevented ventricular fibrillation (VF) in ischemia‐induced canine model of sudden cardiac death (Billman 2009; Bonilla et al. 2012). Therefore, autonomic imbalance and calcium handling abnormality play critical roles in the occurrence of ventricular tachyarrhythmias after MI, and exercise training could ameliorate such pathological conditions. In the failing heart, RyR2 hyperphosphorylation at serine 2814 site by CaMKII increases diastolic calcium leak, leading to delayed after‐depolarization and triggering of VT (Lanner et al. 2010; Gonano et al. 2011). We showed that phosphorylated CaMKII and Ser2814‐phosphorylated RyR2 were significantly increased in MI‐Sed compared with Sham, which were suppressed by exercise. Exercise training was previously reported to reduce RyR2‐induced calcium release from the SR and reduce VT in diabetic mice after MI (Rolim et al. 2015). Thus, inhibition of CaMKII dependent‐RyR2 hyperphosphorylation by chronic exercise training may be a key mechanism underlying the suppression of ventricular arrhythmias in heart failure after MI.

In this study, bisoprolol treatment also restored the hyperphosphorylation of CaMKII and RyR2 at Ser2814, as well as the expression of SERCA2a, suggesting an improvement in calcium handling, as for exercise training. Although β‐blocker treatment did not improve cardiopulmonary function and exercise capacity, it showed a more powerful effect on improving HRV (prolonging R‐R interval and increasing SDNN) and reducing ventricular arrhythmias. Thus, the combination of these two therapies may lead to a better integrative outcome. Vanzelli et al. reported that combined aerobic exercise training and carvedilol treatment had a positive impact on heart failure using a genetic model of sympathetic hyperactivity‐induced heart failure, albeit via a different mechanism (Vanzelli et al. 2013). The beneficial effects of combined therapies, including improved cardiac calcium handling, are mainly related to the effects of exercise training and the reduced myocardial oxidative stress and reversed ventricular remodeling associated with carvedilol therapy (Vanzelli et al. 2013). Our contrasting findings may be attributed to differences in the model, pharmacological agent, and study protocol.

There is now strong evidence for an important role of miRNAs in regulating cardiac function and structural changes during normal cardiac development, as well as in pathological conditions such as heart failure (Bartel 2009; Olson 2014; Pinti et al. 2017). Of more than 1000 miRNAs, miR‐1 and miR‐133 are recognized as muscle‐specific miRNAs and play an important role in the pathogenesis of heart failure and arrhythmia (Li et al. 2015). However, the behavior of miRNAs in heart failure remains controversial. While some studies have reported a reduction in miR‐1 expression in hypertrophy and heart failure (Ikeda et al. 2009; Li et al. 2015; Diniz et al. 2017), others have reported an increase (Matkovich et al. 2009; Long et al. 2012). In this study, miR‐1 expression was upregulated, while miR‐133 was unchanged, in MI groups compared with Sham. The differences in cardiac miR‐1 and miR‐133 expression may depend on the stages and etiology of heart failure. We showed that elevated miR‐1 expression in MI was significantly restored by chronic exercise training and β‐blocker treatment, which is consistent with a previous study showing that β‐adrenergic antagonists can downregulate miR‐1 (Lu et al. 2009). Moreover, some studies reported that exercise training restored cardiac miR‐1 and regulating calcium handling (Melo et al. 2015; Silveira et al. 2017). Terentyev et al. also reported that miR‐1‐overexpressing cardiomyocytes exhibited spontaneous arrhythmogenic oscillations of intracellular calcium, via a selective increase in phosphorylation of the L‐type calcium channel and RyR2 at Serine 2814 (CaMKII‐dependent phosphorylation site), and downstream regulation of PP2A activity (Terentyev et al. 2009).

PP2A is the target molecule of miR‐1(Terentyev et al. 2009), and the authors reported that expression of miR‐1 and miR‐133 were significantly increased in heart failure, while expression of PP2A catalytic and regulatory subunits (putative targets of miR‐133 and miR‐1) were decreased (Belevych et al. 2011). Moreover, the decreased PP2A activity observed in heart failure was accompanied by enhanced CaMKII‐mediated phosphorylation of RyR2 at Ser‐2814, and increased frequency of diastolic calcium waves and after‐depolarizations, in heart failure rats compared with controls (Terentyev et al. 2009). Taken together, these findings suggest that the antiarrhythmic effects of chronic exercise or β‐blocker treatment are, at least in part, related to regulation of miR‐1 expression, and downstream phosphatase activity of calcium handling‐related molecules, in cardiomyocytes.

There are several limitations in this study. Although we analyzed the protein expression levels of calcium handling molecules, we did not measure intracellular calcium content. This is recognized as a major limitation in our study. In addition, the valid sample number in HRV analysis was only 4, and although we showed SDNN value as a major parameter of HRV, the others such as PNN50 were not obtained. We decided to keep 6‐week intervention to obtain enough effectiveness according to the previous researches (Speaker et al. 2014; Rolim et al. 2015). Because structural remodeling process might be almost completed within 1 month after MI or even shorter, we supposed longer intervention would not have yield different results. However, we cannot exclude the possibilities that longer or shorter period of intervention or start period of intervention would yield different results in this study, and further research will be recommended. We do not have baseline echocardiographic data before MI and we didn't check the time‐courses of real‐time PCR, Western blotting, and histological analysis, which became limitations of our study. We used only male mice to avoid the influence of sex difference on the results. It is unclear whether same results were also obtained in female mice because the sex differences are important in post‐MI remodeling.

In summary, we conclude that continuous intensive exercise training can suppress ventricular arrhythmias in subacute to chronic phase of MI through restoring autonomic imbalance and impaired calcium handling, similarly to that for β‐blockers. It is suggested that the antiarrhythmic effects of chronic intensive exercise or β‐blocker treatment are, at least in part, attributed to regulations of miR‐1‐mediated PP2A activity and its downstream targets, hyperphosphorylated CaMKII‐RyR2, in MI. Thus, exercise may be a safe and effective therapy for improving outcome in heart failure patients after MI.

## Conflict of Interest

The authors declare that they have no conflict of interest.
